# Severe Fatigue is Highly Prevalent in Patients with IPF or Sarcoidosis

**DOI:** 10.3390/jcm9041178

**Published:** 2020-04-20

**Authors:** Ada E. M. Bloem, Rémy L. M. Mostard, Naomi Stoot, Jan H. Vercoulen, Jeannette B. Peters, Daisy J. A. Janssen, Jan W. H. Custers, Martijn A. Spruit

**Affiliations:** 1 Institute of Movement Studies, Faculty of Health Care, University of Applied Sciences Utrecht, 3584 CS Utrecht, The Netherlands; jan.custers@hu.nl; 2 ILD Centre of Excellence, Department of Pulmonology, St. Antonius Hospital, 3435 CM Nieuwegein, The Netherlands; 3 Department of Respiratory Medicine, Zuyderland Medical Center Heerlen, 6419 PC Heerlen, The Netherlands; r.mostard@zuyderland.nl (R.L.M.M.); n.stoot@zuyderland.nl (N.S.); 4 Department of Medical Psychology, Radboud University Medical Center, Radboud Institute for Health Sciences, 6525 EZ Nijmegen, The Netherlands; jan.vercoulen@radboudumc.nl (J.H.V.); jeannette.jacobs-peters@radboudumc.nl (J.B.P.); 5 Department of Research and Development, CIRO, 6085 NM Horn, The Netherlands; daisyjanssen@ciro-horn.nl (D.J.A.J.); martijnspruit@ciro-horn.nl (M.A.S.); 6 Department of Health Services Research, Care and Public Health Research Institute, Maastricht University, 6229 ER Maastricht, The Netherlands; 7 Department of Respiratory Medicine, Maastricht University Medical Center, NUTRIM School of Nutrition and Translational Research in Metabolism, 6229 ER Maastricht, The Netherlands; 8 REVAL—Rehabilitation Research Center, BIOMED—Biomedical Research Institute, Faculty of Rehabilitation Sciences, Hasselt University, BE 3500 Diepenbeek, Belgium

**Keywords:** fatigue, interstitial lung disease, idiopathic pulmonary fibrosis, sarcoidosis

## Abstract

In patients with interstitial lung disease (ILD) next to dyspnea, fatigue is expected to be the most prevalent symptom. Surprisingly, the prevalence of severe fatigue has been scarcely studied in ILD patients and limited information on its associated factors is available. This study aimed to determine the prevalence of severe fatigue in patients with idiopathic pulmonary fibrosis (IPF) or pulmonary sarcoidosis and to identify the relationship between fatigue, patient characteristics, and clinical parameters. In this cross-sectional study, fatigue (checklist individual strength-fatigue (CIS-Fat)), demographics, lung function, dyspnea (modified-Medical Research Council (mMRC)), sleepiness (Epworth Sleepiness Scale), anxiety/depression (hospital anxiety and depression scale (HADS-A/HADS-D)), catastrophizing (fatigue catastrophizing scale (FCS)), functional activity impairment (respiratory illness quality-of-life (QoL-RIQ-Activity)), and health status (EuroQol five-dimensional descriptive system (EQ-5D-5L)) were assessed in outpatients with ILD. Mean CIS-Fat scores were 34.1 (SD ± 11.2) in 59 IPF patients and 40.0 (12.3) in 58 sarcoidosis patients. Severe fatigue (SD ± ≥36 points) was present in IPF patients (47.5%) and sarcoidosis (69%). In IPF, CIS-Fat correlated strongly (ρ > 0.5; *p* < 0.01) with FCS, QoL-RIQ-Activity, and EQ-5D-5L-Health and moderately (0.3 < ρ < 0.5; *p* < 0.01) with EQ-5D-5L-Index, mMRC, and HADS-D. In sarcoidosis, CIS-Fat correlated strongly with EQ-5D-5L-Health, QoL-RIQ-Activity, EQ-5D-5L-Index, HADS-D, and mMRC and moderately with FCS and hospitalization <12 months. Severe fatigue is highly prevalent in ILD patients and is associated with dyspnea, depression, catastrophizing, functional activity impairments, and QoL.

## 1. Introduction

Patients with interstitial lung disease (ILD) experience common and distressing respiratory symptoms, e.g., dyspnea on exertion and a cough [1,2]. Moreover, fatigue, which is defined as the subjective feeling of tiredness or exhaustion [3], is assumed to be present and affects ILD patients’ quality of life (QoL) significantly [4,5,6]. 

Two common forms of ILD are idiopathic pulmonary fibrosis (IPF) and sarcoidosis. More than 50% of the patients with IPF reported fatigue as being one of their most significant symptoms, which can manifest in a variety of ways, such as physical fatigue, including muscle fatigue, persistent lack of energy, and overall malaise [7]. The prevalence of severe fatigue in patients with IPF has not been studied extensively. A previous study in patients with sarcoidosis demonstrated that severe fatigue occurred in 47.9% of the patients, of which about two-thirds of the patients had pulmonary sarcoidosis [8]. A similar percentage of severe fatigue was present in patients in clinical remission of sarcoidosis in another study. [9].

The etiology of severe fatigue is poorly understood and likely to be multifactorial. Fatigue is significantly associated with a patient’s health status, reduced physical condition, and functional impairments, irrespective of the degree of lung function impairment [4,10,11,12]. Fatigue may be affiliated by dyspnea [13,14], daytime sleepiness [14,15] (characterized by difficulty staying awake and alert during the day) [16], symptoms of anxiety and/or depression, and fatigue-related catastrophizing [4,7]. Fatigue might be an adverse event of systemic medication used to treat ILD [17,18]. Severe fatigue can have many different causes and is assumed to negatively affect QoL. Severe fatigue will most probably require patient-tailored treatment due to the (combination of) many perpetuating factors [19]. Hence, it is necessary and clinically relevant to study the relationship between severe fatigue and possible underlying factors in patients with ILD.

Therefore, the aims of the present study were (1) to determine the prevalence of severe fatigue in patients with ILD and (2) to evaluate the association between fatigue and clinical parameters.

## 2. Materials and Methods

### 2.1. Study Design and Participants

This cross-sectional prospective clinical study took place in the outpatient clinic of the Department of Respiratory Medicine, Zuyderland Medical Centre Heerlen (The Netherlands) between May 2018 and March 2019. Patients (age ≥18 years) were eligible to participate with a confirmed diagnosis of IPF [2,20] or pulmonary sarcoidosis [21,22], visiting the outpatient department of the chest physician for usual care. Exclusion criteria were insufficient understanding of the Dutch language and/or inability to complete questionnaires because of cognitive impairment or participating at the same time in an intervention study that may have impacted the outcome of this study. Eligible participants were invited by the chest physician (RM) and received a written explanation of the study. Subsequently, the nurse practitioner (NS) informed participants about the research protocol, including instructions to fill in the forms and the data extraction from patient electronic medical records. After giving written informed consent, participants received paper-based questionnaires to be filled out. The study protocol was approved by the Medical Research Ethics Committee of the institution (METCZ20180027) and registered at the Netherlands Trial Register (Trialcode7201).

### 2.2. Measures

#### 2.2.1. Fatigue

Experience of fatigue was assessed by the subscale fatigue of the checklist individual strength-fatigue (CIS-Fat) [23]. The CIS-Fat is a standardized and validated questionnaire that has been used in healthy subjects and among various patient populations [24,25,26]. The CIS-Fat consists of eight items scored on a seven-point Likert scale, with a total range from 8 to 56 points. A score of points ≤26 indicates normal fatigue, between 27–35 mild fatigue, and a score of ≥36 severe fatigue [23,26].

#### 2.2.2. Medical Information

Data extracted from the electronic medical record were: age (years), diagnosis, comorbidities, smoking pack-years, medication, resting transcutaneous oxygen saturation (SpO2; %), oxygen supplementation (yes/no), last available (within the preceding three months) spirometry (forced vital capacity, forced expiratory volume in one second; Liter, % predicted), static lung volumes (total lung capacity, residual volume; Liter, % predicted), and diffusing capacity for carbon monoxide (TLCO; % predicted). [27]. 

#### 2.2.3. Demographic Data 

All participants provided information on their gender (man/woman), height (m), weight (kg), partner (yes/no) and living status (alone/cohabiting), education level (“low educated” meaning maximum preparatory vocational education and “educated” meaning minimal secondary or higher), diagnosis history of the lung disease (years) and hospitalization in the past year (yes/no), working history in the previous two years (yes/no), a history of psychological support (yes/no), smoking status (never, former smoker, current smoker), and the amount of caffeine and alcohol (units per day) consumption. 

#### 2.2.4. Symptom and Limitation Measures

The modified Medical Research Council (mMRC) dyspnea scale was used to classify the severity of dyspnea. The categorizing levels ranged from 0 (“normal”) to 4 (“too breathless to leave the house”) [28,29] Daytime sleepiness was measured by the Epworth Sleepiness Scale (ESS) [30]. The ESS consists of eight questions with a score from 0 (never) to 3 (always), with a total score ranging from 0–24 points; a score of 11 points or higher represents excessive daytime sleepiness (EDS) and normative values are known [31]. From the Quality-of-Life for Respiratory Illness Questionnaire, the general activities of the domain “functional impairment” were assessed (QoL-RIQ/activity) [32]. This activity list contains four questions presenting the impairment in activity due to breathing problems. A higher score in the 7-level score (between “no burden at all” to “a lot of burden”) indicated more impairment. 

#### 2.2.5. Psychological Measures

Symptoms of anxiety and depression were scored using the well-validated Hospital Anxiety and Depression Scale (HADS) [33,34]. This scale is divided into a subscale anxiety (HADS-A) and a subscale depression (HADS-D), both containing seven intermingled items. Each item is rated on a four-point scale, ranging from 0 to 3 points, with 3 points indicating higher symptom frequency. Total scores for each subscale range from 0 to 21 points, categorized as: normal/mild (0–10 points) and moderate/severe (11–21 points, meaning a clinically significant case of anxiety or depression) [33]. 

Catastrophizing has been defined as “an exaggerated negative mental set brought to bear during actual or anticipated painful experience” [35], The fatigue catastrophizing scale (FCS) was used as a measure of fatigue-related catastrophizing. The FCS was modified from the pain catastrophizing scale (PCS) [36,37,38] by replacing the term “pain” with “fatigue” where relevant. The FCS consists of 13 items scored from 0 to 4 points (ranging from 0, “Not at all”, to 4, “All the time”) with a total possible score of 52 points. The higher the score, the more catastrophizing of fatigue was present. The cutoff >30 points has been shown to be associated with clinical relevance [39]. 

The attribution of possible causes from the patient point-of-view was assessed with the causal attribution list (CAL) [40]. The CAL comprises two subscales of “physical” (5 items) and “non-physical” (6 items) causes. Scoring items were divided into “not agree”, “slightly agree”, “strong agree”, and “totally agree”. Item scores were combined into one total score and one score for each of the subscales, whereby a stronger causal attribution was indicated by a higher score. 

#### 2.2.6. Health Status

The EuroQol five-dimensional descriptive system (EQ-5D-5L) comprises two forms; a 5-dimension list (mobility, self-care, usual activities, pain/discomfort, anxiety/depression) with a 5-level classification (no problems, slight, moderate, severe, extreme problems) and the EQ-visual analogue scale (EQ-VAS). The patient was asked to indicate his health state in each dimension (1-digit number). The total result was reported as a 5-digit number that describes the patient’s health state. The digit number IIIII indicated no problems in each dimension. Additionally, the results were converted into an index value (EQ-5D-5L-Index). The EQ-VAS recorded the patient’s self-rated health on a vertical visual analogue scale, where the endpoints were labelled from 0 (“worst imaginable health”) to 100 points (“best imaginable health”) [41,42,43].

### 2.3. Statistical Analyses

Statistical analysis was performed using IBM SPSS Statistics (Version 25). Patients’ characteristics were presented with appropriate measures of central tendency and dispersion. Numerical data were tested for normality by a mean-median ratio, SD-mean ratio, and judging histogram [44]. Differences between groups for continuous data were analyzed by an unpaired t-test or the non-parametric pendant (Mann–Whitney U test) where appropriate. Categorical data were analyzed with the Chi-square or Fisher Exact test. A *p*-value of ≤0.05 was considered as statistically significant. Correlations were calculated by Pearson’s r or when the assumptions were violated by Spearman’s rho. In case of missing values, cases were excluded pairwise. The range for what constitutes a weak, moderately strong, strong, or very strong correlation was respectively 0.1 ≤ r < 0.3, 0.3 ≤ r < 0.5, 0.5 ≤ r <0.7, and 0.7 ≤ r < 1.0 [45,46] (level of significance *p* < 0.05). A multivariable model was conducted to assess the associations between the dependent variable QoL-RIQ/activity and independent in univariate analysis significant variables (*p* < 0.01). In case of multicollinearity, identified variables (variance inflation factor (VIF) > 5) were removed from the model [47]. 

## 3. Results

### 3.1. Patient Characteristics

A total of 121 Patients with ILD volunteered to participate (92% of invited patients with IPF, 42% of invited patients with sarcoidosis). Four participants were excluded due to the absence of a fully completed CIS-Fat questionnaire. Participants (*n* = 117) were diagnosed with IPF (*n* = 59, 50%), of which 26% were classified as a severe disease (forced vital capacity (FVC) <50% and/or TLCO <40% predicted [48]) or sarcoidosis (*n* = 58, 50%). In this ILD sample, there was a male predominance (62%), the median age was 66 years, and the mean body mass index (BMI) was slightly elevated (27.6 kg/m2). Overall, the patients were well educated (64% secondary level education or higher), had a partner (74%), and a smoking history (59%; 8.5 pack-years). A total of 50% reported a daily coffee consumption of ≥3 cups per day, and 42% reported ≥1 units of daily alcohol consumption. Most patients had one or more comorbidity but had no differences after stratification for degree of fatigue severity. For detailed differences between patients with IPF or sarcoidosis see Table 1 and Table 2.

### 3.2. Prevalence of Severe Fatigue

Patients with ILD (*n* = 117) had a mean CIS-Fat score of 37.0 (SD12.1) points, of which 58% had severe fatigue (CIS-Fat ≥36 points, *n* = 68). Patients with IPF had a significantly lower mean CIS-Fat score (34.1 points, SD11.2) compared to patients with sarcoidosis (40 points, SD12.3) (*p* < 0.01). In addition, severe fatigue was prevalent in 48% of patients with IPF (*n* = 28) and in 69% of patients with sarcoidosis (*n* = 40) (Table 1).

### 3.3. Factors Associated with Severe Fatigue in Patients with IPF

**Figure 1:**
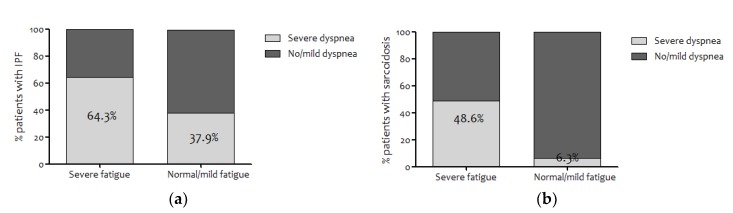
(**a**) Prevalence of patients with idiopathic pulmonary fibrosis (IPF) with no/mild dyspnea (Modified-Medical Research Council (mMRC) <2) or severe dyspnea (mMRC ≥2) after stratification for the degree of fatigue (checklist individual strength-fatigue (CIS-Fat) mild/moderate <36 and severe >35). (**b**) Prevalence of patients with sarcoidosis with no/mild dyspnea (mMRC <2) or severe dyspnea (mMRC ≥2) after stratification for the degree of fatigue (CIS-Fat mild/moderate <36 and severe >35).

The group of IPF patients with severe fatigue had a significantly higher proportion of men (89.3% vs. 64.5%), higher daily coffee consumption (71.4% vs. 40.0% ≥3 cups a day), lower mean diffusion capacity (45.0% vs. 53.0% TLCO % predicted), and reported more dyspnea (64.3% vs. 37.9% mMRC grade ≥2) (Figure 1a) compared to IPF patients with normal/mild fatigue (Table 1). Spirometry results were not related to fatigue severity (Table 1). The depression score on the HADS (5.0 vs. 3.0 points) and the result of thr FCS (21.0 vs. 5.0 points) were different between patients with and without severe fatigue, respectively. Patients with severe fatigue versus normal/mild fatigue felt more impaired in functional activities (15.0 vs. 11.0 points QoL-RIQ/activity), and QoL scores were lower (0.67 vs. 0.80 points EQ-5D-5L-Index, 55.0 vs. 71.0 points EQ-5D-5L-Health-today; indication IIIII was not reported) (Table 3).

### 3.4. Factors Associated with Severe Fatigue in Patients with Sarcoidosis

Patients with sarcoidosis and severe fatigue compared to those with sarcoidosis and normal/mild fatigue were significantly (*p* < 0.05) less educated (30% vs. 0% school level “low”), had visited a psychologist more often (42.5% vs. 11.1%), and had a higher average of smoking pack-years (5.7 vs. 0.9 pack-years), but no significant differences in lung function results were present (Table 1). Sarcoidosis patients with severe fatigue reported significantly more severe dyspnea (49.0% vs. 6.3% mMRC) (Figure 1b) and excessive sleepiness (21.1% vs. 0% EDS) (Table 2). Anxiety and depression scores were significantly higher in sarcoidosis patients with severe fatigue compared to those with normal-to-mild fatigue (5.0 vs. 3.0 points HADS-A, 6.0 vs. 1.0 points HADS-D). In addition, scores on catastrophizing were significantly higher (11.5 vs. 4.0 points FCS). Moreover, patients with severe fatigue versus normal/mild fatigue felt more impaired in activities (15.0 vs. 6.0 points QoL-RIQ/activity), and QoL scores were lower (0.68 vs. 0.89 points EQ-5D-5L-Index, 55.3 vs. 81.2 points EQ-5D-5L-Health-today; indication IIIII was reported by 8% of the patients).

### 3.5. Correlations of Fatigue 

Significant correlations (moderately strong or strong) with the CIS-Fat score were found in patients with IPF for drinking coffee (cups a day) (r = 0.269; *p* < 0.05), forced expiratory volume in one second (FEV1)% predicted (r = −0.329; *p* < 0.05), TLCO% predicted (r = −0.324; *p* < 0.05), mMRC (ρ = 0.374; *p* < 0.01), HADS-A (ρ = 0.281; *p* < 0.05), HADS-D (ρ = 0.369; *p* < 0.01), FCS (ρ = 0.572; *p* < 0.01), QoL-RIQ/activity (ρ = 0.544; *p* < 0.01), EQ-5D-5L-Index value (ρ = −0.414; *p* < 0.01), EQ-5D-5L-Health today (ρ = −0.529; *p* < 0.01). 

In patients with sarcoidosis from significant moderate to strong correlations with the CIS-Fa score were found with hospitalization <12 months (ρ = 0.359; *p* < 0.01), mMRC (ρ = 0.535; *p* < 0.01), ESS (ρ = 0.282; *p* < 0.05), HADS-A (ρ = 0.264; *p* < 0.05), HADS-D (ρ = 0.556; *p* < 0.01), FCS (ρ = 0.481; *p* < 0.01), CAL-physical (ρ = 0.392; *p* < 0.01), QoL-RIQ/activity (ρ = 0.604; *p* < 0.01), EQ-5D-5L-Index value (ρ = −0.577; *p* < 0.01), EQ-5D-5L-Health today (ρ = −0.710; *p* < 0.01) were found.

### 3.6. Determinants of Functional Impairment QoL-RIQ/Activity

The following significant correlations (*p* < 0.01) with the QoL-RIQ/activity for patients with IPF or sarcoidosis were identified: mMRC (ρ = 0.608; ρ = 0.667), HADS-D (ρ = 0.582; ρ = 0.611), FCS (ρ = 0.639; ρ = 0.533), CIS-Fat (ρ = 0.544; ρ = 0.604), causal attribution list (CAL)-physical (ρ = 0.490; ρ = 0.650).

The stepwise multiple regression model in IPF explained 65.7% of variance in QoL-RIQ/activity (adjusted R2 = 0.657; Std Error of the Estimate 3.245; *p* < 0.01), whereby significant predictors were: FCS (50.1%), CIS-Fa (10.1%), and mMRC (5.5%) (regression equation QoL-RIQ/activity = 2.438 + 0.161*FCS + 0.182*CIS-Fat (scale) + 1.403* mMRC).

In sarcoidosis, multiple regression modelling explained 66.8% of variance in QoL-RIQ/activity (adjusted R2 0.67, Std Error of the Estimate 3.533; *p* < 0.01), with the significant predictors mMRC (43.7%), CAL-Physical (17.4%), and FCS (5.7%) (regression equation QoL-RIQ/activity = −0.086 + 1.965*mMRC + 3.620*CAL-Physical + 0.156*FCS).

## 4. Discussion

This study clearly shows that severe fatigue was present in a substantial proportion of patients with ILD; in 48% of patients with IPF and 69% of patients with pulmonary sarcoidosis. Furthermore, fatigue was significantly associated (ρ > 0.3) with dyspnea, depression, fatigue-related catastrophizing, activity impairments, and quality-of-life.

### 4.1. Severe Fatigue in Patients with IPF or Sarcoidosis

Severe fatigue has been observed frequently in patients with chronic obstructive pulmonary disease (COPD) (ranging between 41 to 75%) [49,50,51], asthma (62%) [52], or cancer survivors (range from 7% to 52%) [53]. The current findings of severe fatigue of 48% and 69% in patients with IPF or sarcoidosis, respectively, fit well. This is significantly higher compared with elderly non-COPD subjects, of which 10% report severe fatigue [54,55]. Interestingly, severe fatigue was more prevalent in the sarcoidosis patients than in the IPD patients, despite the fact that the sarcoidosis group consisted of fewer men, was younger of age, was higher educated, had worked the last two years, had more psychological support, had more never smokers, and had less comorbidities.

### 4.2. Factors Associated with Fatigue in Patients with IPF or Sarcoidosis

Overall, the sarcoidosis patients had a mildly impaired lung function, which did not correlate with fatigue. Previous findings in patients with sarcoidosis [56], COPD [50], or asthma [52] showed that the forced vital capacity was not different between patients with and without severe fatigue, suggesting that the degree of lung function impairment does not play a major role in the development and/or maintenance of severe fatigue. In contrast, FEV1% predicted and TLCO% predicted were moderately correlated with CIS-Fat in the IPF patients, and severely fatigued patients had a significant lower diffusion capacity (*p* < 0.05) vs. no/mild fatigue. Sheth et al. [57] found in patients with IPF that a low diffusion capacity and a higher fatigue score were independent predictors of frailty. Frailty was not assessed in the current study. However, these data may suggest that patients with IPF with a low diffusion capacity and who report severe fatigue are at risk of becoming frail. 

Similar to patients with COPD, more patients with IPF or sarcoidosis with severe fatigue experienced severe dyspnea (mMRC ≥ 2) than those with normal/mild fatigue [13]. Indeed, in IPF, severe dyspnea was present in 64% of the patients with severe fatigue and in 49% of the patients with sarcoidosis. 

Although the terms “fatigue” and “sleepiness” are often used interchangeably, both phenomena are distinct [58]. Bosse-Henck [14] studied excessive daytime sleepiness (defined as ESS ≥ 16) and severe fatigue (Fatigue Assessment Scale ≥35 points) in sarcoidosis patients and found sleepiness and/or fatigue in 27% of their sample. In the current study, ESS values were not different to the normative value [31], although EDS (ESS > 10) was more present in sarcoidosis patients with severe fatigue (21%) than in IPF patients with severe fatigue (5%). However, severe fatigue was obviously more present in both groups (58%) compared to EDS (8%), which suggests poor sleep quality may not be the main driver of severe fatigue in ILD patients.

It is known that in patients with chronic respiratory diseases, symptoms of anxiety and depression are common [59,60]. In the current study, the patients with severe fatigue had significantly higher anxiety (sarcoidosis) and depression (IPF, sarcoidosis) scores vs. normal/mild fatigue. These findings affirmed the findings of respectively 12% and 7% in a tertiary referral clinic for ILD [60]. 

The current study shows that catastrophizing is moderate to strong, correlated to fatigue, which is a novel finding. Indeed, the relation of catastrophizing to fatigue has not been investigated before in patients with ILD. Catastrophizing may contribute to increased intensity of symptom experience [35] and, in chronic pain, catastrophizing is a prospective marker for the risk of severe disability [61]. Catastrophizing can influence the experience of fatigue and seems to be a good predictor of fatigue severity [62]. Catastrophizing might be of great importance to consider, because if patients avoid activity by negative attention, this might decrease their physical functioning. Moreover, patients with IPF or sarcoidosis experienced high subjective impairments in activity and significantly more in those patients with severe fatigue. 

Although objective measurements of functional capacity in this study are lacking, different aspects (both cognitive as perceptive) were pointed out to play an important role of this experienced impairment in activity. Indeed, dyspnea (IPF, sarcoidosis), fatigue (IPF), fatigue-related catastrophizing (IPF, sarcoidosis), and causal attributions scores (sarcoidosis) explained 66% (IPF) and 67% (sarcoidosis) of the variance of the perceived impairment in activity (QoL/RIQ-activity). 

### 4.3. Limitations and Strength

This study had several methodological considerations. First, the sample group of patients with ILD was restricted to patients with IPF and pulmonary sarcoidosis only, and only patients were included who were visiting the outpatient consultation of an ILD-specialized pulmonologist (RM). This may limit the external validity of the current findings. The reason of the lower response rate on the invitation letter of patients with sarcoidosis (42%) vs. patients with IPF (92%) is unknown. Second, as is known, predictors of physical activity are the exercise capacity and fatigue in patients with ILD [56,63], but the study setting and funding did not allow us to assess physical functioning, such as a 6 min walk distance and peak aerobic capacity. Therefore, it remains unknown whether and to what extent a lower level of physical functioning may explain, at least partially, the presence of severe fatigue in patients with ILD. Third, in patients with COPD only receiving usual care, the proportion of patients with severe fatigue doubles over a period of 4 years [49]. The current cross-sectional study design did not allow us to assess possible changes in fatigue over time. Fourth, the generic EQ-5D-5L is not an ILD-specific questionnaire for QoL and consequently it does not capture disease-specific effects of ILD. However, the EQ-5D-5L is validated in patients with different lung diseases (COPD, ILD) and will make comparisons between patients with different lung diseases and the general population possible [64,65].

The strength of the study was the sample size of 117 patients with IPF or sarcoidosis. Next to dyspnea, severe fatigue has now been indicated highly prevalent in patients with IPF or sarcoidosis. A broad range of patient characteristics, psychological, behavioral, and health factors, including dyspnea, sleepiness, anxiety, depression, fatigue-related catastrophizing, functional impairment, and quality-of-life, were collected, which provided unique new insights into severe fatigue. For clinical management, it is important to know about this.

Finally, fatigue is a multidimensional phenomenon and just a part of all possible associated factors were investigated, consequently a patient-tailored treatment advice to reduce fatigue based on this study is not possible. Longitudinal prospective studies, including patient characteristics, psychological aspects, and physical functioning, are required for a better understanding of severe fatigue in IPF and sarcoidosis and to explore its long-term impact on quality-of-life.

## 5. Conclusions

To conclude, fatigue is an important symptom in patients with ILD, and ILD patients with severe fatigue experienced more severe dyspnea, sleepiness, anxiety, depression, fatigue-related catastrophizing, functional activity impairments, and a lower QoL. In clinical management of patients with ILD, it is recommended to assess fatigue, catastrophizing thoughts, and causal attributions of fatigue, because these elements together with dyspnea are related to the functional impairments in activities of the patients.

## Figures and Tables

**Table 1:** General characteristics of patients with interstitial lung disease, idiopathic pulmonary fibrosis (IPF), and sarcoidosis, stratified for fatigue severity in normal/mild or severe fatigue based on the checklist individual strength-fatigue (CIS-Fat) questionnaire.

Variables	Patients with ILD (Total Group)	Patients with IPF	Fatigue Severity in IPF Patients			Patients with Sarcoidosis	Fatigue Severity in Sarcoidosis Patients			IPF—Sarcoidosis
			Normal/Mild Fatigue <36 *p*	Severe Fatigue Fatigue ≥36 *p*	*p*-Value		Normal/Mild Fatigue <36 *p*	Severe Fatigue Fatigue ≥36 *p*	*p*-Value	*p*-Value
*n* (%)	117	59 (50.4)	31 (52.5)	28 (47.5)		58 (49.6)	18 (31.0)	40 (69.0)		
CIS-Fat	37.0 ± 12.1	34.1 ± 11.2	25.5 ± 7.2	43.5 ± 5.8	*p* < 0.01	40.0 ± 12.3	24.9 ± 8.5	46.7 ± 6.2	*p* < 0.01	*p* < 0.01
General Characteristics										
Gender (male, %)	73 (62.4)	45 (76.3)	20 (64.5)	25 (89.3)	*p* < 0.05*	28 (48.3)	12 (66.7)	16 (40.0)	ns	*p* < 0.01*
Age (years, IQR)	66.0 (53.5–74)	73.0(70.0–78.0)	72.0 (70.0–77.0)	73.0 (70.3–78.8)	ns	53.5 (45.8–62.0)	55.5 (48.8–66.0)	51.5 (43.8–59.8)	ns	*p* < 0.01
Weight (kg)	82.2 ± 14.7	81.4 ± 14.8	82.7 ± 16.1	80.0 ± 13.4	ns	83.0 ± 14.6	84.5 ± 9.5	82.3 ± 16.5	ns	ns
BMI ^a^ (kg/m2)	27.6 ± 4.2	27.6 ± 4.1	28.1 ± 4.4	27.0 ± 3.8	ns	27.6 ± 4.2	27.2 ± 3.1	27.7 ± 4.7	ns	ns
Partner (*n*, %)	86 (73.5)	43 (72.9)	23 (74.2)	20 (71.4)	ns	43 (74.1)	15 (83.3)	28 (70.0)	ns	ns
Living together (*n*, %)	80 (68.4)	39 (66.1)	21 (67.7)	18 (64.3)	ns	41 (70.7)	15 (83.3)	26 (65.0)	ns	ns
Education ^b^, ≥secondary level (*n*, %)	73 (63.5)	27 (47.4)	13 (41.9)	14 (53.8)	ns	46 (79.3)	18 (100.0)	28 (70.0)	*p* < 0.05^#^	*p* < 0.01
Diagnosis time ≤1 year (*n*, %)	36 (31.3)	21 (36.2)	12 (40.0)	9 (32.1)	ns	15 (26.3)	4 (23.5)	11 (27.5)	ns	ns
Hospitalization ≤1 year (*n*, %)	24 (20.7)	14 (23.7)	7 (22.6)	7 (25.0)	ns	10 (17.5)	1 (5.6)	9 (22,5)	ns	ns
Work last 2 years (*n*, %)	48 (41.0)	12 (20.3)	7 (22.6)	5 (17.9)	ns	36 (62.1)	13 (72.2)	23 (57.5)	ns	*p* < 0.01
Psychological support (*n*, %)	27 (23,1)	8 (13.6)	5 (16.1)	3 (10.7)	ns	19 (32.8)	2 (11.1)	17 (42.5)	*p*<0.05^#^	*p* < 0.05
Smoking ^a^ current/former (*n*, %)	68 (58.6)	46 (78.0)	23 (74.2)	23 (82.1)	ns**	22 (38.6)	5 (29.4)	17 (42.5)	ns**	*p* < 0.05**
Pack-years^+^ (*n*)	8.5 ± 15.1	13.5 ± 18.1	12.2 ± 16.4	15.1 ± 20.3	ns	4.0 ± 9.8	0.9 ± 2.6	5.7 ± 11.8	*p* < 0.05	*p* < 0.01
Coffee^++ b^ cup ≥3 (*n*, %)	58 (50.4)	32 (55.2)	12 (40.0)	20 (71.4)	*p* < 0.05*	26 (45.6)	9 (52.9)	17 (42.5)	ns	ns
Alcohol^++ b^ glass ≥1 (*n*, %)	48 (41.7)	24 (41.4)	12 (40.0)	12 (42.9)	ns	24 (42.1)	9 (52.9))	15 (37.5)	ns	ns
Spirometry, static lung volumes, and diffusing capacity										
TLC ^e^ (liter)	5.2 ± 1.4	4.6 ± 1.1	4.4 ± 1.1	4.7 ± 1.1	ns	6.0 ± 1.3	6.3 ± 1.2	5.8 ± 1.3	ns	*p* < 0.01
TLC ^f^ (% predicted)	85.3 ± 20.8	73.3 ± 14.3	72.6 ± 12.9	74.1 ± 16.0	ns	98.6 ± 18.8	96.5 ± 12.6	99.6 ± 21.2	ns	*p* < 0.01
RV ^d^ (liter)	1.8 ± 0.5	1.6 ± 0.4	1.5 ± 0.4	1.7 ± 0.3	ns	2.0 ± 0.6	2.0 ± 0.6	2.0 ± 0.5	ns	*p* < 0.01
RV (% predicted)	80.3 ± 26.6	64.4 ± 13.8	63.3 ± 14.5	65.6 ± 13.3	ns	98.0 ± 26.2	90.8 ± 18.0	101.3 ± 28.9	ns	*p* < 0.01
FVC (liter)	3.4 ± 1.1	2.9 ± 0.8	2.8 ± 0.8	3.0 ± 0.9	ns	3.9 ± 1.1	4.1 ± 1.1	3.8 ± 1.1	ns	*p* < 0.01
FVC (% predicted)	90.7 ± 21.6	83.2 ± 19.6	83.5 ± 18.7	82.9 ± 20.8	ns	98.2 ± 21.1	100.9 ± 16.3	97.0 ± 23.1	ns	*p* < 0.01
FEV_1_ (liter)	2.6 ± 0.8	2.3 ± 0.6	2.3 ± 0.7	2.3 ± 0.6	ns	3.0 ± 0.9	3.1 ± 0.9	2.9 ± 0.9	ns	*p* < 0.01
FEV_1_ (% predicted)	89.5 ± 20.7	87.3 ± 20.9	89.3 ± 22.1	84.9 ± 19.5	ns	91.9 ± 20.5	94.1 ± 17.6	90.9 ± 21.8	ns	ns
TLCO ^c^ (liter)	5.5 ± 2.4	3.9 ± 1.3	4.2 ± 1.5	3.6 ± 1.0	ns	7.3 ± 2.0	7.8 ± 2.0	7.1 ± 2.0	ns	*p* < 0.01
TLCO ^d^ (% predicted)	65.1 ± 23.2	49.1 ± 14.7	53.0 ± 15.4	45.0 ± 12.8	*p* < 0.05	82.2 ± 18.0	86.6 ± 17.2	80.1 ± 18.2	ns	*p* < 0.01
Comorbidities										
Comorbidity (*n*, %)					ns**				ns**	*p* < 0.05**
none	44 (37.6)	15 (25.4)	9 (29.0)	6 (21.4)		29 (50.0)	10 (55.6)	19 (47.5)		
1	42 (35.9)	25 (42.4)	13 (41.9)	12 (42.9)		17 (29.3)	3 (16.7)	14 (35.0)		
>1	31 (26.5)	19 (32.2)	9 (29.0)	10 (35.7)		12 (20.7)	5 (27.8)	7 (17.5)		
Medication										
IPF: antifibrotic (*n*, %)		51 (86.4)	27 (87.1)	24 (85.7)		0 (0.0)	0 (0.0)	0 (0.0)		*p* < 0.01*
Nintenadib (*n*, %)		17 (33.3)	9 (33.3)	8 (33.3)	ns					
Pirfenidon (*n*, %)		34 (66.7)	18 (66.7)	16 (66.7)	ns					
Immunosuppressant^+++^ (*n*, %)	27 (23.1)	5 (8.5)	0 (0.0)	5 (17.9)	*p* < 0.05^#^	22 (37.9)	5 (27.8)	17 (42.5)	ns	*p* < 0.01*
Heart rate-lowering medication (*n*, %)	23 (19.7)	15 (25.4)	9 (29.0)	6 (21.4)	ns	8 (13.8)	3 (16.7)	5 (12.5)	ns	ns
Antidepressant medication (*n*, %)	6 (5.1)	5 (8.5)	1 (3.2)	4 (14.3)	ns	1 (1.7)	0 (0.0)	1 (2.5)	ns	ns
Antihypertensive medication (*n*, %)	37 (31.6)	25 (42.4)	16 (51.6)	9 (32.1)	ns	12 (20.7)	4 (22.2)	8 (20.0)	ns	*p* < 0.05*
Other medication for pulmonary conditions (*n*, %)	38 (32.5)	7 (11.9)	2 (6.5)	5 (17.9)	ns	31 (53.4)	7 (38.9)	24 (60.0)	ns	*p* < 0.01*

Data is presented as mean ± SD, median (IQR) or number (%). *p*-value in bold indicates *p* < 0.05: * Pearson Chi-square; ^#^ Fisher’s exact test; ** Mann–Whitney U Test. ^+^ Pack-year, number of years smoking × average number of cigarettes smoked per day/20. ^++^ number of consumed cups/glasses a day. ^+++^ Immunosuppressant including prednisone (corticosteroids). Alphabetic characters in superscript indicates a sample size deviant from *n* = 117, in the order: ^a^ *n* = 116, ^b^ *n* = 115, ^c^ *n* = 112, ^d^ *n* = 110, ^e^ *n* = 109, ^f^ *n* = 108,. Abbreviations: arbitrary units (au); body mass index (BMI; kg/m2); checklist individual strength (CIS); forced expiratory volume in one second (FEV1); forced vital capacity (FVC); interstitial lung disease (ILD); included (incl.); idiopathic pulmonary fibrosis (IPF); interquartile range (IQR); number of subjects (*n*); not significant (ns); residual volume (RV); residual volume to total lung capacity ratio (RV/TLC Ratio); total lung capacity (TLC); transfer factor of the lung for carbon monoxide (measured in ml/min/mm Hg) (TLCO).

**Table 2:** Comorbidities of patients with idiopathic pulmonary fibrosis (IPF) or sarcoidosis, stratified for fatigue severity.

Variables	Patients with ILD (Total Group)	Patients with IPF	Fatigue Severity in IPF Patients			Patients with Sarcoidosis	Fatigue Severity in Sarcoidosis Patients			IPF—Sarcoidosis
			Normal/Mild Fatigue <36 *p*	Severe Fatigue Fatigue ≥36 *p*	*p*-Value		Normal/Mild Fatigue <36 *p*	Severe Fatigue Fatigue ≥36 *p*	*p*-Value	*p*-Value
*n* (%)	117	59 (50.4)	31 (52.5)	28 (47.5)		58 (49.6)	18 (31.0)	40 (69.0)		
CIS (*p*, 8–56)	37.0 ± 12.1	34.1 ± 11.2	25.5 ± 7.2	43.5 ± 5.8	*p* < 0.01	40.0 ± 12.3	24.9 ± 8.5	46.7 ± 6.2	*p* < 0.01	*p* < 0.01
General Characteristics										
Comorbidity										
Comorbidity (*n*, %)					ns**				ns**	*p* < 0.05**
none	44 (37.6)	15 (25.4)	9 (29.0)	6 (21.4)		29 (50.0)	10 (55.6)	19 (47.5)		
1	42 (35.9)	25 (42.4)	13 (41.9)	12 (42.9)		17 (29.3)	3 (16.7)	14 (35.0)		
>1	31 (26.5)	19 (32.2)	9 (29.0)	10 (35.7)		12 (20.7)	5 (27.8)	7 (17.5)		
Comorbidity ≥1 (*n*, %)	73 (62.4)	44 (74.6)	22 (71.0)	22 (78.6)	ns*	29 (50.0)	8 (44.4)	21 (52.5)	ns*	*p* < 0.01*
Comorbidities										
Hypertension (*n*, %)	22 (18.8)	17 (28.8)	9 (29.0)	8 (28.6)	ns*	5 (8.6)	1 (5.6)	4 (10.0)	ns^#^	*p* < 0.01*
Pulmonary hypertension (*n*, %)	2 (1.7)	2 (3.4)	0 (0.0)	2 (7.1)	ns^#^	0 (0.0)	0 (0.0)	0 (0.0)	-	*n* s^#^
COPD/asthma (*n*, %)	6 (5.1)	0 (0.0)	0 (0.0)	0 (0.0)	-	6 (10.3)	1 (5.6)	5 (12.5)	ns^#^	*p* <0.05^#^
Cardiac failure (*n*, %)	12 (10.3)	6 (10.2)	3 (9.7)	3 (10.7)	ns^#^	6 (10.3)	4 (22.2)	2 (5.0)	ns^#^	ns*
Cardiac sarcoidosis (*n*, %)	1 (0.9)	0 (0.0)	0 (0.0)	0 (0.0)	-	1 (1.7)	1 (5.6)	0 (0.0)	ns^#^	ns^#^
Cardiac surgery CABG and/or Heart Valve (*n*, %)	8 (6.8)	6 (10.2)	4 (12.9)	2 (7.1)	ns^#^	2 (3.4)	1 (5.6)	1 (2.5)	ns^#^	ns^#^
Diabetes Mellitus (*n*, %)	12 (10.3)	8 (13.6)	3 (9.7)	5 (17.9)	ns^#^	4 (6.9)	2 (11.1)	2 (5.0)	ns^#^	ns^#^
OSAS (*n*, %)	4 (3.4)	3 (5.1)	1 (3.2)	2 (7.2)	ns^#^	1 (1.7)	1 (5.6)	0 (0.0)	ns^#^	ns^#^
Eyes-Uveitis (*n*, %)	5 (4.3)	0 (0.0)	0 (0.0)	0 (0.0)	-	5 (8.6)	2 (11.1)	3 (7.5)	ns^#^	*p* <0.05^#^
TIA/CVA (*n*, %)	7 (6.0)	5 (8.5)	1 (3.2)	4 (14.3)	ns^#^	2 (3.4)	1 (5.6)	1 (2.5)	ns^#^	ns^#^
Carotid artery stenosis/sPAD (*n*, %)	6 (5.1)	5 (8.5)	2 (6.5)	3 (10.7)	ns^#^	1 (1.7)	0 (0.0)	1 (2.5)	ns^#^	ns^#^
Other comorbidities (*n*, %)	36 (30.8)	20 (33.9)	13 (41.9)	7 (25.0)	ns*	16 (27.6)	4 (22.2)	12 (30.0)	ns^#^	ns*

Data is presented as mean ± SD, number (%). *p*-value in bold indicates a significant difference: * Pearson Chi-square; ^#^ Fisher’s exact test; ** Mann–Whitney U Test. Abbreviations: coronary artery bypass grafting (CABG); checklist individual strength (CIS); chronic obstructive pulmonary disease (COPD); cerebrovascular accident (CVA); idiopathic pulmonary fibrosis (IPF); interquartile range (IQR); number of subjects (*n*); obstructive sleep apnea syndrome (OSAS); symptomatic peripheral arterial disease (sPAD); transient ischemic attack (TIA).

**Table 3:** Questionnaire results of patients with interstitial lung disease, idiopathic pulmonary fibrosis (IPF), and sarcoidosis, stratified for fatigue severity.

Variables	Patients with ILD (Total Group)	Patients with IPF	Fatigue Severity in IPF Patients			Patients with Sarcoidosis	Fatigue Severity in Sarcoidosis Patients			IPF—Sarcoidosis
			Normal/Mild Fatigue <36 *p*	Severe Fatigue Fatigue ≥36 *p*	*p*-Value		Normal/Mild Fatigue <36 *p*	Severe Fatigue Fatigue ≥36 *p*	*p*-Value	*p*-Value
*n* (%)	117	59 (50.4)	31 (52.5)	28 (47.5)		58 (49.6)	18 (31.0)	40 (69.0)		
CIS (p, 8–56)	37.0 ± 12.1	34.1 ± 11.2	25.5 ± 7.2	43.5 ± 5.8	*p* < 0.01	40.0 ± 12.3	24.9 ± 8.5	46.7 ± 6.2	*p* < 0.01	*p* < 0.01
Dyspnea										
mMRC-Dyspnea grade ^g^ range 0–4 (p, IQR)	1.0 (1.0–2.0)	2.0 (1.0–3.0)	1.0 (1.0–3.0)	2.0 (1.0–3.0)	ns**	1.0 (1.0–2.0)	0.5 (0.0–1.0)	1.0 (1.0–2.0)	*p* < 0.05**	*p* < 0.05**
mMRC ^g^ grade ≥2 (moderate-severe dyspnea) (*n*, %)	48 (43.6)	29 (50.9)	11 (37.9)	18 (64.3)	*p* < 0.05	19 (35.8)	1 (6.3)	18 (48.6)	*p* < 0.05	ns
Sleepiness										
ESS ^i^ (p, IQR, 0–24)	5.0 (4.0–8.0)	5.0 (4.0–8.0)	4.0 (3.0–7.0)	5.5 (4.0–8.3)	ns	5.0 (4.0–8.0)	5.0 (3.8–6.0)	6.5 (3.8–10.0)	ns	ns
ESS > 10 excessive ^i^ (*n*, %)	9 (8.4)	1 (2.0)	0	1 (4.5)	ns	8 (14.3)	0	8 (21.1)	*p* < 0.05^#^	*p* < 0.05^#^
Anxiety and Depression										
HADS anxiety ^e^ range 0–21 (p, IQR)	5.0 (2.0–7.8)	5.0 (2.0–8.0)	4.0 (2.0–7.8)	6.0 (4.0–8.0)	ns	4.0 (2.0–7.0)	3.0 (2.0–5.3)	5.0 (3.0–8.0)	*p* < 0.05**	ns
(HADS anxiety ≥11points) ^e^ (*n*, %)	12 (10.7)	5 (9.1)	2 (7.1)	3 (11.1)	ns	7 (12.3)	2 (11.1)	5 (12.8)	ns	ns
HADS depression ^c^ range 0–21 (p, IQR)	4.5 (2.0–7.0)	5.0 (2.0–7.0)	3.0 (2.0–6.5)	5.0 (4.0–8.0)	*p* < 0.05**	4.0 (1.0–7.0)	1.0 (1.0–3.5)	6.0 (3.0–8.0)	*p* < 0.01**	ns
(HADS depression ≥11 points) ^c^ (*n*, %)	9 (7.9)	5 (8.9)	2 (6.9)	3 (11.1)	ns	4 (6.9)	0	4 (10.0)	ns	ns
Fatigue-related Catastrophizing										
FCS ^f^ (p, IQR)	11.0 (3.0–23.0)	11.0 (2.0–26)	5.0 (0.3–14.5)	21.0 (11.0–28.5)	*p* < 0.01**	10.0 (3.8–18.0)	4.0 (0.0–11.5)	11.5 (6.5–23.0)	*p* < 0.01**	ns
FCS ^f^ grade >30 (*n*, %)	13 (11,7)	7 (13.2)	2 (7.1)	5 (20)	ns	6 (10.3)	0	6 (15.0)	ns	ns
Causal Attributions of Fatigue										
CAL^g^ Sum score (p, 11–44)	18.3 ± 5.1	18.4 ± 4.5	17.8 ± 4.5	19.2 ± 4.5	ns	18.3 ± 5.6	17.6 ± 6.8	18.6 ± 5.0	ns	ns
CAL Physical Sum ^d^ (p, 5–20)	9.4 ± 3.4	9.7 ± 3.1	9.1 ± 3.0	10.4 ± 3.1	ns	9.1 ± 3.7	7.9 ± 4.0	9.6 ± 3.5	ns	ns
CAL Non-Physical Sum ^e^ (p, 6–24)	8.9 ± 2.9	8.6 ± 2.4	8.7 ± 2.6	8.5 ± 2.1	ns	9.2 ± 3.3	9.6 ± 4.1	9.0 ± 3.0	ns	ns
Quality of Life Respiratory Illness (Functional Impairment)										
QoL-RIQ/activity ^h^ (p, IQR)	13.0 (8.0–17.0)	14.0 (10.3–17.0)	11.0 (8.0–15.0)	15.0 (13.0–20.0)	*p* < 0.01**	10.5 (7.0–16.8)	6.0 (4.0–9.0)	15.0 (9.0-18.0)	*p* < 0.01**	*p* < 0.05**
Quality of Life, Health Status										
EQ-5D-5L ^a^, index values (p, 0–1)	0.74 ± 0.20	0.74 ± 0.18	0.80 ± 0.16	0.67 ± 0.17	*p* < 0.01	0.75 ± 0.23	0.89 ± 0.14	0.68 ± 0.23	*p* < 0.01	ns
EQ-5D-5L^b^, VAS (p, 0–100)	63.1 ± 18.1	63.3 ± 16.5	71.0 ± 15.6	55.0 ± 13.4	*p* < 0.01	63.0 ± 19.7	81.2 ± 11.5	55.3 ± 17.2	*p* < 0.01	ns

Data is presented as mean ± SD, median (IQR), or number (%). *p*-value in bold indicates a significant difference: * Pearson Chi-square; ^#^ Fisher’s exact test; ** Mann–Whitney U Test. Alphabetic characters in superscript indicate a sample size deviant from *n* = 117, in the order: ^a^ *n* = 116, ^b^ *n* = 115, ^c^ *n* = 114, ^d^ *n* = 113, ^e^ *n* = 112, ^f^ *n* = 111, ^g^ *n* = 110, ^h^ *n* = 108, ^i^ *n* = 107. ^1^ IIIII. ^2^ Answer option “not applicable” is out of score. Abbreviations: Acceptance of Disease and Impairments Questionnaire (ADIQ); checklist individual strength (CIS); EuroQol, 5 dimensions, 5 levels (standardized measure of health status) (EQ-5D-5L); Epworth Sleepiness Scale (ESS); fatigue catastrophizing scale (FCS); hospital anxiety and depression scale (HADS); interstitial lung disease (ILD); included (incl.); idiopathic pulmonary fibrosis (IPF); interquartile range (IQR); modified Medical Research Council (mMRC) dyspnoea scale; number of subjects (*n*); points (p); Quality of Life for Respiratory Illness Questionnaire (QoL-RIQ/activity), domain “functional activity impairment”, list “general activities”.

## References

[1] Bradley B., Branley H.M., Egan J.J., Greaves M.S., Hansell D.M., Harrison N.K., Hirani N., Hubbard R., Lake F., Millar A.B. (2008). Interstitial lung disease guideline: The British Thoracic Society in collaboration with the Thoracic Society of Australia and New Zealand and the Irish Thoracic Society. Thorax.

[2] Travis W.D., Costabel U., Hansell D.M., King T.E., Lynch D.A., Nicholson A.G., Ryerson C.J., Ryu J.H., Selman M., Wells A.U. (2013). An official American Thoracic Society/European Respiratory Society statement: Update of the international multidisciplinary classification of the idiopathic interstitial pneumonias. Am. J. Respir. Crit. Care Med..

[3] Landmark-Hoyvik H., Reinertsen K.V., Loge J.H., Kristensen V.N., Dumeaux V., Fossa S.D., Borresen-Dale A.L., Edvardsen H. (2010). The genetics and epigenetics of fatigue. PM R..

[4] Swigris J.J., Brown K.K., Abdulqawi R., Buch K., Dilling D.F., Koschel D., Thavarajah K., Tomic R., Inoue Y. (2018). Patients’ perceptions and patient-reported outcomes in progressive-fibrosing interstitial lung diseases. Eur. Respir. Rev..

[5] Atkins C., Wilson A.M. (2017). Managing fatigue in sarcoidosis—A systematic review of the evidence. Chron. Respir. Dis..

[6] Sharma O.P. (1999). Fatigue and sarcoidosis. Eur. Respir. J..

[7] U.S. Food and Drug Administration’s A series of reports from the U.S. Food and Drug Administration’s (FDA’s) Patient-Focused Drug Development Initiative. Proceedings of the Idiopathic Pulmonary Fibrosis Public Meeting.

[8] Voortman M., Hendriks C.M.R., Elfferich M.D.P., Bonella F., Moller J., De Vries J., Costabel U., Drent M. (2019). The Burden of Sarcoidosis Symptoms from a Patient Perspective. Lung.

[9] Korenromp I.H.E., Heijnen C.J., Vogels O.J.M., van den Bosch J.M.M., Grutters J.C. (2011). Characterization of chronic fatigue in patients with sarcoidosis in clinical remission. Chest.

[10] Michielsen H.J., Drent M., Peros-Golubicic T., De Vries J. (2006). Fatigue is associated with quality of life in sarcoidosis patients. Chest.

[11] Holland A.E., Hill C.J., Conron M., Munro P., McDonald C.F. (2008). Short term improvement in exercise capacity and symptoms following exercise training in interstitial lung disease. Thorax.

[12] Swigris J.J., Fairclough D.L., Morrison M., Make B., Kozora E., Brown K.K., Wamboldt F.S. (2011). Benefits of pulmonary rehabilitation in idiopathic pulmonary fibrosis. Respir. Care.

[13] Kentson M., Todt K., Skargren E., Jakobsson P., Ernerudh J., Unosson M., Theander K. (2016). Factors associated with experience of fatigue, and functional limitations due to fatigue in patients with stable COPD. Ther. Adv. Respir. Dis..

[14] Bosse-Henck A., Koch R., Wirtz H., Hinz A. (2017). Fatigue and Excessive Daytime Sleepiness in Sarcoidosis: Prevalence, Predictors, and Relationships between the Two Symptoms. Respiration.

[15] Cho J., Teoh A., Roberts M., Wheatley J. (2019). The prevalence of poor sleep quality and its associated factors in patients with interstitial lung disease: A cross-sectional analysis. ERJ Open Res..

[16] Ito E., Inoue Y. (2015). The International Classification of Sleep Disorders, third edition. American Academy of Sleep Medicine. Includes bibliographies and index. Nihon Rinsho..

[17] Fleischer M., Hinz A., Brahler E., Wirtz H., Bosse-Henck A. (2014). Factors associated with fatigue in sarcoidosis. Respir. Care.

[18] Oltmanns U., Kahn N., Palmowski K., Trager A., Wenz H., Heussel C.P., Schnabel P.A., Puderbach M., Wiebel M., Ehlers-Tenenbaum S. (2014). Pirfenidone in idiopathic pulmonary fibrosis: Real-life experience from a German tertiary referral center for interstitial lung diseases. Respiration.

[19] Spruit M.A., Vercoulen J.H., Sprangers M.A.G., Wouters E.F.M. (2017). FAntasTIGUE consortium Fatigue in COPD: An important yet ignored symptom. Lancet Respir. Med..

[20] Raghu G., Collard H.R., Egan J.J., Martinez F.J., Behr J., Brown K.K., Colby T.V., Cordier J.F., Flaherty K.R., Lasky J.A. (2011). An official ATS/ERS/JRS/ALAT statement: Idiopathic pulmonary fibrosis: Evidence-based guidelines for diagnosis and management. Am. J. Respir. Crit. Care Med..

[21] Hunninghake G.W., Costabel U., Ando M., Baughman R., Cordier J.F., du Bois R., Eklund A., Kitaichi M., Lynch J., Rizzato G. (1999). ATS/ERS/WASOG statement on sarcoidosis. American Thoracic Society/European Respiratory Society/World Association of Sarcoidosis and other Granulomatous Disorders. Sarcoidosis Vasc. Diffuse Lung Dis..

[22] Zhou Y., Lower E.E., Li H., Baughman R.P. (2016). Clinical management of pulmonary sarcoidosis. Expert Rev. Respir. Med..

[23] Vercoulen J.H., Swanink C.M., Fennis J.F., Galama J.M., van der Meer J.W., Bleijenberg G. (1994). Dimensional assessment of chronic fatigue syndrome. J. Psychosom. Res..

[24] Beurskens A.J., Bultmann U., Kant I., Vercoulen J.H., Bleijenberg G., Swaen G.M. (2000). Fatigue among working people: Validity of a questionnaire measure. Occup. Environ. Med..

[25] Bultmann U., de Vries M., Beurskens A.J., Bleijenberg G., Vercoulen J.H., Kant I. (2000). Measurement of prolonged fatigue in the working population: Determination of a cutoff point for the checklist individual strength. J. Occup. Health Psychol..

[26] Worm-Smeitink M., Gielissen M., Bloot L., van Laarhoven H.W.M., van Engelen B.G.M., van Riel P., Bleijenberg G., Nikolaus S., Knoop H. (2017). The assessment of fatigue: Psychometric qualities and norms for the Checklist individual strength. J. Psychosom. Res..

[27] Quanjer P.H., Tammeling G.J., Cotes J.E., Pedersen O.F., Peslin R., Yernault J.C. (1994). Lung volumes and forced ventilatory flows. Work Group on Standardization of Respiratory Function Tests. European Community for Coal and Steel. Official position of the European Respiratory Society. Rev. Mal. Respir..

[28] Mahler D.A., Rosiello R.A., Harver A., Lentine T., McGovern J.F., Daubenspeck J.A. (1987). Comparison of clinical dyspnea ratings and psychophysical measurements of respiratory sensation in obstructive airway disease. Am. Rev. Respir. Dis..

[29] Mahler D.A., Wells C.K. (1988). Evaluation of clinical methods for rating dyspnea. Chest.

[30] Johns M.W. (1991). A new method for measuring daytime sleepiness: The Epworth sleepiness scale. Sleep.

[31] Sander C., Hegerl U., Wirkner K., Walter N., Kocalevent R.D., Petrowski K., Glaesmer H., Hinz A. (2016). Normative values of the Epworth Sleepiness Scale (ESS), derived from a large German sample. Sleep Breath.

[32] Maille A., Koning C., Zwinderman A., Willems L., Dijkman J., Kaptein A. (1997). The development of the ‘Quality-of-life for Respiratory Illness Questionnaire (QOL-RIQ)’: A disease-specific quality-of-life questionnaire for patients with mild to moderate chronic non-specific lung disease. Respir. Med..

[33] Zigmond A.S., Snaith R.P. (1983). The hospital anxiety and depression scale. Acta Psychiatr. Scand..

[34] Bjelland I., Dahl A.A., Haug T.T., Neckelmann D. (2002). The validity of the Hospital Anxiety and Depression Scale: An updated literature review. J. Psychosom. Res..

[35] Sullivan M.J., Thorn B., Haythornthwaite J.A., Keefe F., Martin M., Bradley L.A., Lefebvre J.C. (2001). Theoretical perspectives on the relation between catastrophizing and pain. Clin. J. Pain.

[36] Sullivan M.J., Bishop S.R., Pivik J. (1995). The pain catastrophizing scale: Development and validation. Psychol. Assess..

[37] Osman A., Barrios F.X., Kopper B.A., Hauptmann W., Jones J., O’Neill E. (1997). Factor structure, reliability, and validity of the Pain Catastrophizing Scale. J. Behav. Med..

[38] Osman A., Barrios F.X., Gutierrez P.M., Kopper B.A., Merrifield T., Grittmann L. (2000). The Pain Catastrophizing Scale: Further psychometric evaluation with adult samples. J. Behav. Med..

[39] Sullivan M.J.L. (2009). PCS: Pain Catastrophizing Scale: User manual. Montreal: Departments of Psychology, Medicine, and Neurology, School of Physical and Occupational Therapy.

[40] Vercoulen J.H., Swanink C.M., Galama J.M., Fennis J.F., Jongen P.J., Hommes O.R., van der Meer J.W., Bleijenberg G. (1998). The persistence of fatigue in chronic fatigue syndrome and multiple sclerosis: Development of a model. J. Psychosom. Res..

[41] Herdman M., Gudex C., Lloyd A., Janssen M., Kind P., Parkin D., Bonsel G., Badia X. (2011). Development and preliminary testing of the new five-level version of EQ-5D (EQ-5D-5L). Qual. Life Res..

[42] Janssen M.F., Pickard A.S., Golicki D., Gudex C., Niewada M., Scalone L., Swinburn P., Busschbach J. (2013). Measurement properties of the EQ-5D-5L compared to the EQ-5D-3L across eight patient groups: A multi-country study. Qual. Life Res..

[43] van Reenen M., Janssen B. (2015). EQ-5D-5L User Guide: Basic Information on How to Use the EQ-5D-5L Instrument.

[44] Altman D.G. (1990). Practical Statistics for Medical Research.

[45] Beurskens S. (2008). Meten in de Praktijk.

[46] Cohen J. (1988). Statistical Power Analysis for the Behavioral Sciences.

[47] Akinwande M.O., Dikko H.G., Samson A. (2015). Variance inflation factor: As a condition for the inclusion of suppressor variable (s) in regression analysis. Open J. Stat..

[48] Egan J.J., Martinez F.J., Wells A.U., Williams T. (2005). Lung function estimates in idiopathic pulmonary fibrosis: The potential for a simple classification. Thorax.

[49] Peters J.B., Heijdra Y.F., Daudey L., Boer L.M., Molema J., Dekhuijzen P.N., Schermer T.R., Vercoulen J.H. (2011). Course of normal and abnormal fatigue in patients with chronic obstructive pulmonary disease, and its relationship with domains of health status. Patient Educ. Couns..

[50] Goertz Y.M.J., Spruit M.A., Van ’t Hul A.J., Peters J.B., Van Herck M., Nakken N., Djamin R.S., Burtin C., Thong M.S.Y., Coors A. (2019). Fatigue is highly prevalent in patients with COPD and correlates poorly with the degree of airflow limitation. Ther. Adv. Respir. Dis..

[51] Van Herck M., Antons J., Vercoulen J.H., Goertz Y.M.J., Ebadi Z., Burtin C., Janssen D.J.A., Thong M.S.Y., Otker J., Coors A. (2019). Pulmonary Rehabilitation Reduces Subjective Fatigue in COPD: A Responder Analysis. J. Clin. Med..

[52] Van Herck M., Spruit M.A., Burtin C., Djamin R., Antons J., Goertz Y.M.J., Ebadi Z., Janssen D.J.A., Vercoulen J.H., Peters J.B. (2018). Fatigue is Highly Prevalent in Patients with Asthma and Contributes to the Burden of Disease. J. Clin. Med..

[53] Abrahams H.J., Gielissen M.F., Schmits I.C., Verhagen C.A., Rovers M.M., Knoop H. (2016). Risk factors, prevalence, and course of severe fatigue after breast cancer treatment: A meta-analysis involving 12 327 breast cancer survivors. Ann. Oncol..

[54] Baghai-Ravary R., Quint J.K., Goldring J.J., Hurst J.R., Donaldson G.C., Wedzicha J.A. (2009). Determinants and impact of fatigue in patients with chronic obstructive pulmonary disease. Respir. Med..

[55] Engberg I., Segerstedt J., Waller G., Wennberg P., Eliasson M. (2017). Fatigue in the general population- associations to age, sex, socioeconomic status, physical activity, sitting time and self-rated health: The northern Sweden MONICA study 2014. BMC Public Health.

[56] Strookappe B., De Vries J., Elfferich M., Kuijpers P., Knevel T., Drent M. (2016). Predictors of fatigue in sarcoidosis: The value of exercise testing. Respir. Med..

[57] Sheth J.S., Xia M., Murray S., Martinez C.H., Meldrum C.A., Belloli E.A., Salisbury M.L., White E.S., Holtze C.H., Flaherty K.R. (2019). Frailty and geriatric conditions in older patients with idiopathic pulmonary fibrosis. Respir. Med..

[58] Neu D., Linkowski P., Le Bon O. (2010). Clinical complaints of daytime sleepiness and fatigue: How to distinguish and treat them, especially when they become ‘excessive’ or ‘chronic’?. Acta Neurol. Belg..

[59] Maurer J., Rebbapragada V., Borson S., Goldstein R., Kunik M.E., Yohannes A.M., Hanania N.A. (2008). ACCP Workshop Panel on Anxiety and Depression in COPD Anxiety and depression in COPD: Current understanding, unanswered questions, and research needs. Chest.

[60] Holland A.E., Fiore J.F., Bell E.C., Goh N., Westall G., Symons K., Dowman L., Glaspole I. (2014). Dyspnoea and comorbidity contribute to anxiety and depression in interstitial lung disease. Respirology.

[61] Solomon B.K., Wilson K.G., Henderson P.R., Poulin P.A., Kowal J., McKim D.A. (2015). A Breathlessness Catastrophizing Scale for chronic obstructive pulmonary disease. J. Psychosom. Res..

[62] Lukkahatai N., Saligan L.N. (2013). Association of catastrophizing and fatigue: A systematic review. J. Psychosom. Res..

[63] Bahmer T., Kirsten A.M., Waschki B., Rabe K.F., Magnussen H., Kirsten D., Gramm M., Hummler S., Brunnemer E., Kreuter M. (2016). Clinical Correlates of Reduced Physical Activity in Idiopathic Pulmonary Fibrosis. Respiration.

[64] Nolan C.M., Longworth L., Lord J., Canavan J.L., Jones S.E., Kon S.S., Man W.D. (2016). The EQ-5D-5L health status questionnaire in COPD: Validity, responsiveness and minimum important difference. Thorax.

[65] Szentes B.L., Kreuter M., Bahmer T., Birring S.S., Claussen M., Waelscher J., Leidl R., Schwarzkopf L. (2018). Quality of life assessment in interstitial lung diseases: A comparison of the disease-specific K-BILD with the generic EQ-5D-5L. Respir. Res..

